# Supplementary material for: The Moderating Role of Self-Control and Financial Strain in the Relation between Exposure to the Food Environment and Obesity: The GLOBE Study
Source: Int J Environ Res Public Health. 2019 Feb 25;16(4):674. doi: 10.3390/ijerph16040674 (PMC6406643; doi:10.3390/ijerph16040674)
Supplement: Supplementary file 1 [file ijerph-16-00674-s001.zip › ijerph-434833-suppl xml.docx]

**Table S1.** Associations between measures of the food environment and body weight using alternative buffer sizes.

| **Exposure Variables** | **Body Mass Index** | **Weight Status** | | |
| --- | --- | --- | --- | --- |
|  |  | **Normal Weight** | **Overweight** | **Obesity** |
|  | **B (95% CI)** | **RRR (95% CI)** | **RRR (95% CI)** | **RRR (95% CI)** |
| Density of fast food outlets in a 250 m buffer | **−0.03** (−0.05; −0.00) | Ref. | 0.99 (0.98; 1.01) | 0.98 (0.95; 1.00) |
| Density of all food outlets in a 250 m buffer | **−0.01** (−0.01; −0.00) | Ref. | **1.00** (0.99; 1.00) | 1.00 (0.99; 1.00) |
| Density of fast food outlets in an 800 m buffer | −0.04 (−0.10; 0.02) | Ref. | 0.98 (0.95; 1.01) | 0.98 (0.94; 1.03) |
| Density of all food outlets in an 800 m buffer | −0.01 (−0.01; 0.00) | Ref. | 1.00 (0.99; 1.00) | 1.00 (0.99; 1.01) |
|  |  |  |  |  |

Note: Ref. = Reference category. B = regression coefficient. RRR = Relative Risk Ratio. All analyses are adjusted for age, gender, education, children in the household, household equivalent income, employment status, country of birth and length of residency. Bold values represent statistically significant associations as defined by the 95% confidence interval.

**Table S2.** Associations between measures of the food environment and body weight using quartiles of food outlet density.

| **Exposure Variables** | **Body Mass Index** | **Weight Status** | | |
| --- | --- | --- | --- | --- |
|  |  | **Normal Weight** | **Overweight** | **Obesity** |
|  | **B (95% CI)** | **RRR (95% CI)** | **RRR (95% CI)** | **RRR (95% CI)** |
| Density of fast food outlets in a 400 m buffer |  |  |  |  |
| Quartile 1 (lowest density) | Ref. | Ref. | Ref. | Ref. |
| Quartile 2 | 0.01 (−0.54; 0.57) | Ref. | 0.93 (0.71; 1.22) | 1.06 (0.75; 1.51) |
| Quartile 3 | −0.18 (−0.70; 0.34) | Ref. | 1.01 (0.77; 1.32) | 0.93 (0.65; 1.33) |
| Quartile 4 1 (highest density) | **−0.54** (−1.05; −0.04) | Ref. | 0.91 (0.70; 1.19) | **0.67** (0.46; 0.99) |
| Density of all food outlets in a 400 m buffer |  |  |  |  |
| Quartile 1 1 (lowest density) | Ref. | Ref. | Ref. | Ref. |
| Quartile 2 | 0.02 (−0.52; 0.56) | Ref. | 1.08 (0.83; 1.41) | 0.98 (0.69; 1.39) |
| Quartile 3 | 0.11 (−0.45; 0.68) | Ref. | 0.98 (0.74; 1.29) | 1.08 (0.75; 1.56) |
| Quartile 4 1 (highest density) | −0.45 (−0.95; 0.04) | Ref. | 0.91 (0.70; 1.19) | 0.70 (0.48; 1.01) |

Note: Ref. = Reference category. B = regression coefficient. RRR= Relative risk ratio. All analyses are adjusted for age, gender, education, children in the household, household equivalent income, employment status, country of birth and length of residency. Bold values represent statistically significant associations as defined by the 95% confidence interval.
